# Immunomodulatory Strategies for Spinal Cord Injury

**DOI:** 10.26717/bjstr.2022.45.007202

**Published:** 2022-07-29

**Authors:** Jonghyuck Park

**Affiliations:** Pharmaceutical Sciences, College of Pharmacy and Spinal Cord and Brain Injury Research Center, University of Kentucky, USA

**Keywords:** Spinal Cord Injury, Nerve Regeneration, Immunomodulation, Gene Therapy, Nanomedicine, Stem Cell

## Abstract

Neuroinflammation is a key secondary event after spinal cord injury (SCI) and can increase barriers to regeneration, leading to various neurological disorders. Infiltrated hematogenous innate immune cells into the injured site are considered the main effector cells of the inflammatory responses after SCI. Glucocorticoids were the standard of care for spinal cord trauma for years due to their anti-inflammatory properties yet were also associated with unwanted side effects. While the administration of glucocorticoids is controversial, immunomodulatory strategies that limit inflammatory responses provide the potential therapeutic approaches to promote functional regeneration following SCI. Herein, we will discuss emerging therapeutic strategies to modulate inflammatory responses to enhance nerve recovery after spinal cord trauma.

## Introduction

Traumatic Spinal Cord Injury (SCI) results in an initial primary injury, followed by secondary events including ischemia, anoxia, and excitotoxicity for the first minutes, hours, and days after injury [1–4]. As a key secondary event, inflammation is initiated in part by the rapid influx of circulating immune cells such as neutrophils, inflammatory monocytes, and macrophages [5–7]. These cells begin to release various inhibitory factors which contribute to an inhibitory microenvironment and damage adjacent intact tissue, thereby inducing permanent loss of locomotor and somatosensory functions below the level of injured spinal cord [8–10]. Although inflammatory response induces additional secondary damages, it is necessary to trigger wound healing and tissue regeneration after injury [5,11]. Additionally, there can be unwanted results such as systemic infection or disease when depleting immune cell populations. Therefore, systemic suppression of immune responses is unlikely to provide long-term therapeutic effects for SCI treatment. This review will focus on therapeutic strategies that reprogram the pro-inflammatory microenvironment towards a pro-regenerative milieu to limit inflammation-mediated secondary injury and promote functional regeneration after SCI.

## Gene Therapy

Various gene therapy-based approaches have been introduced in the last twenty years and continue to be utilized as a powerful therapeutic approach to alter or correct defective genes for the treatment of multiple diseases [12,13]. While direct delivery of biologics is effective for short-term dosage, gene therapy provides the long-term sustained expression of the gene of interest. Vectors for gene therapy can be broadly classified as non-viral and viral vectors14. Non-viral vectors are cost-effective and less immunogenic, however, their ability to transfect cells is limited particularly in vivo [14]. Viral vectors can overcome these limitations by incorporating their genetic information into the host cell’s genome, inducing greater transduction efficacy and long-term expression of target genes. Choosing an appropriate vector will be the most important factor to achieve immunomodulatory therapy for SCI. The timing of onset of delivered transgene expression can be critical. The transgene expression by herpes simplex virus (HSV) or adenovirus occurs within 24 hours after delivery, which is faster than other viral vectors [15]. The transgene expression by lentivirus (LV) exhibits within 48 hours after administration and peaks the expression level of transgene after 3 days [6,16,17]. Vector size also can be an important factor since retrovirus can carry a maximum of 10 kb of single-stranded RNA and adeno-associated virus (AAV) can contain less than 5 kb of single-stranded DNA. Adenovirus and HSV can contain relatively higher transgene capacities. Viral vectors can be immunogenic compared to non-virial vectors which can result in unwanted side effects. While adenovirus can cause significant inflammatory responses, LV and AAV are considered less immunogenic and exhibit a transient immune response after administration [18].

## Nanoparticles

Nanomedicine employing polymeric nanoparticle (NP) has received significant attention due to its inherent therapeutic potential to modulate immune responses to cure inflammatory responses-mediated disorders including SCI [19–22]. Previous study utilizing poly(lactide-coglycolide) (PLG)-based NPs with a negative surface charge indicated that intravenously administered NPs distracted circulating immune cells such as neutrophils and inflammatory monocytes prior to extravasation into the injured spinal cord, thereby reducing the pathological system indirectly after SCI [22]. NPs with highly negative zeta potential are taken up by targeted circulating immune cells through the scavenger receptors such as macrophage receptor with collagenous structure (MARCO) and reprogram them to affect their migration to the spleen [23]. Furthermore, some NP-positive cells accumulate at the injury, enabling upregulation of the expression of pro-regenerative factors that directly promote a more permissive environment after SCI [24,25]. Additionally, locally delivered poly (2-hydroxyethyl methacrylate) (PHEMA) and polycaprolactone (PCL) based NPs have been shown to modulate immune responses by attenuating the activation of pro-inflammatory macrophages and microglia specifically after SCI [26]. Other NPs including polystyrene [27], poly (lactic acid) [28], iron oxide [29], and gold [30] have also been employed to reprogram immune cells to modulate inflammatory responses after injury.

## Stem Cell Therapy

Stem cells can proliferate and differentiate into any cell type present in the body. Considering their therapeutic potential and abilities of self-renewal and differentiation, stem cells are widely used following SCI [31,32]. In a preclinical study, the transplantation of mouse embryonic stem cells (ESC) into a rat spinal cord after SCI, demonstrated differentiation of transplanted stem cells into astrocytes, oligodendrocytes and neurons improving hindlimb weight support and coordination [33]. In a clinical study, bone-marrow derived mesenchymal stem cell transplanted patients showed improvement in American Spinal Injury Association Impairment Scale (AIS) grade from A to B in SCI patients [34]. Adult stem cells are more susceptible to immune rejection compared to ESC since they express major histocompatibility complex (MHC)-II and CD86 [35]. However, adult stem cells can reprogram the inflammatory environment by releasing multiple anti-inflammatory cytokines [36]. The neural stem cells (NSC) in the spinal cord have the ability to reprogram the immune cells after SCI [37]. They promote macrophage polarization towards a more pro-regenerative phenotype enhancing the expression of anti-inflammatory factors to improve nerve regeneration. NSC release multiple factors after an injury such as TGF-b, prostaglandin E, and nitric oxide that increase the number of regulatory T cells enhancing the expression of pro-regenerative factors. In addition, NSC can directly contact T cells and increase regulatory T cell populations, thereby enhancing the expression of anti-inflammatory factors while limiting the expression of pro-inflammatory factors [37]. Mesenchymal stem cells (MSC) can also modulate the inflammatory responses after SCI. MSC secrete interleukin 1 receptor antagonist (IL1-RA) that can induce the polarization of macrophages into pro- regenerative phenotype [38]. In addition, MSC-mediated over-expression of IL-6 and hepatocyte growth factor (HGF) can influence monocytes and induce the secretion of the high level of pro-regenerative factors including IL-10 in the injured site for immunomodulation [39]. Moreover, delivered MSC into the injured spinal cord limited the infiltration of the circulating inflammatory subset of immune cells to the injured site and promoted the phenotypical changes of macrophages and microglia into a more anti- inflammatory phenotype. MSC also restored the broken blood spinal cord barrier to prevent additional damage, thereby enhancing functional recovery after SCI [39].

## Conclusion and Future Directions

Immunotherapeutic approaches for the treatment of SCI have the capacity to indirectly and/or directly enhance functional recovery. Although many preclinical studies have demonstrated therapeutic effects of immunomodulatory factors on SCI, the introduction of these strategies in the clinic faces various limitations for SCI victims. Further investigations will be required to assess how each of the immunomodulatory factors can be employed synergistically to reprogram the immune system to promote functional recovery after SCI while limiting life-threatening side effects.

## Abbreviations:

HGF: Hepatocyte Growth Factor
MSC: Mesenchymal Stem Cells
ESC: Embryonic Stem Cells
MARCO: Macrophage Receptor with Collagenous Structure
PCL: Polycaprolactone
AAV: Adeno-Associated Virus
SCI: Spinal Cord Injury

## References

[1] Due Michael R, Park Jonghyuck, Zheng Lingxing, Walls Michael, Allette Yohance M, (2014) Acrolein involvement in sensory and behavioral hypersensitivity following spinal cord injury in the rat. J Neurochem 128: 776–786. 24147766 10.1111/jnc.12500 PMC3947461

[2] Park Jonghyuck, Lim Eunjeong, Back Seungkeun, Na Heungsik, Park Yongdoo, (2010) Nerve regeneration following spinal cord injury using matrix metalloproteinase- sensitive, hyaluronic acid-based biomimetic hydrogel scaffold containing brain-derived neurotrophic factor. J Biomed Mater Res A 93: 1091–1099. 19768787 10.1002/jbm.a.32519

[3] You G, He Z (2006) Glial inhibition of CNS axon regeneration. Nat Rev Neurosci 7: 617–627. 16858390 10.1038/nrn1956 PMC2693386

[4] Park Jonghyuck, Zheng Lingxing, Acosta Glen, Vega-Alvarez Sasha, Chen Zhe, (2015) Acrolein contributes to TRPA1 up-regulation in peripheral and central sensory hypersensitivity following spinal cord injury. J Neurochem 135: 987–997. 26365991 10.1111/jnc.13352 PMC4715756

[5] Donnelly DJ, Popovich PG (2008) Inflammation and its role in neuroprotection, axonal regeneration and functional recovery after spinal cord injury. Exp Neurol 209: 378–388. 17662717 10.1016/j.expneurol.2007.06.009 PMC2692462

[6] Park Jonghyuck, Decker Joseph T, Smith Dominique R, Cummings Brian J, Anderson Aileen J, (2018) Reducing inflammation through delivery of lentivirus encoding for anti- inflammatory cytokines attenuates neuropathic pain after spinal cord injury. J Control Release 290: 88–101. 30296461 10.1016/j.jconrel.2018.10.003 PMC6240362

[7] Chen Zhe, Park Jonghyuck, Butler Breanne, Acosta Glen, Vega-Alvarez Sasha, (2016) Mitigation of sensory and motor deficits by acrolein scavenger phenelzine in a rat model of spinal cord contusive injury. Journal of Neurochemistry 138: 328–338. 27060873 10.1111/jnc.13639 PMC4936922

[8] Okada S (2016) The pathophysiological role of acute inflammation after spinal cord injury. Inflamm Regen 36: 20. 29259693 10.1186/s41232-016-0026-1 PMC5725917

[9] Park J, Muratori B, Shi R (2014) Acrolein as a novel therapeutic target for motor and sensory deficits in spinal cord injury. Neural Regen Res 9: 677–683. 25206871 10.4103/1673-5374.131564 PMC4146266

[10] Park Jonghyuck, Zheng Lingxing, Marquis Andrew, Walls Michael, Duerstock Brad, (2014) Neuroprotective role of hydralazine in rat spinal cord injury-attenuation of acrolein-mediated damage. J Neurochem 129: 339–349. 24286176 10.1111/jnc.12628 PMC3980042

[11] David S, Kroner A (2011) Repertoire of microglial and macrophage responses after spinal cord injury. Nat Rev Neurosci 12: 388–399. 21673720 10.1038/nrn3053

[12] Simonato Michele, Bennett Jean, Boulis Nicholas M, Castro Maria G, Fink David J, (2013) Progress in gene therapy for neurological disorders. Nat Rev Neurol 9: 277–291. 23609618 10.1038/nrneurol.2013.56 PMC3908892

[13] Verma IM, Somia N (1997) Gene therapy - promises, problems and prospects. Nature 389: 239–242. 9305836 10.1038/38410

[14] Nayerossadat N, Maedeh T, Ali PA (2012) Viral and nonviral delivery systems for gene delivery. Adv Biomed Res 1: 27. 23210086 10.4103/2277-9175.98152 PMC3507026

[15] Blits B, Bunge MB (2006) Direct gene therapy for repair of the spinal cord. J Neurotrauma 23: 508–520. 16629633 10.1089/neu.2006.23.508

[16] Park Jonghyuck, Decker Joseph T, Margul Daniel J, Smith Dominique R, Cummings Brian J, (2018) Local Immunomodulation with Anti-inflammatory Cytokine-Encoding Lentivirus Enhances Functional Recovery after Spinal Cord Injury. Mol Ther 26: 1756–1770. 29778523 10.1016/j.ymthe.2018.04.022 PMC6037204

[17] Margul Daniel J, Park Jonghyuck, Boehler Ryan M, Smith Dominique R, Johnson Mitchell A, (2016) Reducing neuroinflammation by delivery of IL-10 encoding lentivirus from multiple-channel bridges. Bioeng Transl Med 1: 136–148. 27981242 10.1002/btm2.10018 PMC5125399

[18] Nayak S, Herzog RW (2010) Progress and prospects: immune responses to viral vectors. Gene Ther 17: 295–304. 19907498 10.1038/gt.2009.148 PMC3044498

[19] Ventola CL (2017) Progress in Nanomedicine: Approved and Investigational Nanodrugs. P T 42: 742–755. 29234213 PMC5720487

[20] Landesman-Milo D, Peer D (2012) Altering the immune response with lipid-based nanoparticles. J Control Release 161: 600–608. 22230342 10.1016/j.jconrel.2011.12.034

[21] Danhier Fabienne, Ansorena Eduardo, Silva Joana M, Coco Régis, Le Breton Aude, (2012) PLGA-based nanoparticles: an overview of biomedical applications. J Control Release 161: 505–522. 22353619 10.1016/j.jconrel.2012.01.043

[22] Park Jonghyuck, Zhang Yining, Saito Eiji, Gurczynski Steve J, Moore Bethany B, (2019) Intravascular innate immune cells reprogrammed via intravenous nanoparticles to promote functional recovery after spinal cord injury. Proc Natl Acad Sci USA 116: 14947–14954. 31285339 10.1073/pnas.1820276116 PMC6660718

[23] Getts DR, Shea LD, Miller SD, King NJ (2015) Harnessing nanoparticles for immune modulation. Trends Immunol 36: 419–427. 26088391 10.1016/j.it.2015.05.007 PMC4603374

[24] Van Rooijen N, Van Kesteren-Hendrikx E (2012) Clodronate liposomes: perspectives in research and therapeutics. J Liposome Res 12: 81–94. 10.1081/lpr-120004780 12604042

[25] Geremia Nicole M, Bao Feng, Rosenzweig Trina E, Hryciw Todd, Weaver Lynne, (2012) CD11d Antibody Treatment Improves Recovery in Spinal Cord-Injured Mice. J Neurotrauma 29: 539–550. 22044160 10.1089/neu.2011.1976 PMC4853192

[26] Papa Simonetta, Rossi Filippo, Ferrari Raffaele, Mariani Alessandro, De Paola Massimiliano, (2013) Selective nanovector mediated treatment of activated proinflammatory microglia/macrophages in spinal cord injury. ACS Nano 7: 9881–9895. 24138479 10.1021/nn4036014

[27] Maldonado Roberto A, La Mothe Robert A, Ferrari Joseph D, Zhang Ai-Hong, Rossi Robert J, (2015) Polymeric synthetic nanoparticles for the induction of antigen-specific immunological tolerance. Proc Natl Acad Sci USA 112: E156–165. 25548186 10.1073/pnas.1408686111 PMC4299193

[28] Saito Eiji, Kuo Robert, Kramer Kevin R, Gohel Nishant, Giles David A, (2019) Design of biodegradable nanoparticles to modulate phenotypes of antigen- presenting cells for antigen-specific treatment of autoimmune disease. Biomaterials 222: 119432. 31480002 10.1016/j.biomaterials.2019.119432 PMC6830542

[29] Pal Ajay, Singh Anand, Nag Tapas C, Chattopadhyay Parthaprasad, Mathur Rashmi, (2013) Iron oxide nanoparticles and magnetic field exposure promote functional recovery by attenuating free radical-induced damage in rats with spinal cord transection. Int J Nanomedicine 8: 2259–2272. 23818782 10.2147/IJN.S44238 PMC3693820

[30] Hutter Eliza, Boridy Sebastien, Labrecque Simon, Lalancette-Hébert Melanie, Kriz Jasna, (2010) Microglial response to gold nanoparticles. ACS Nano 4: 2595–2606. 20329742 10.1021/nn901869f

[31] Muheremu A, Peng J, Ao Q (2016) Stem cell-based therapies for spinal cord injury. Tissue Cell 48: 328–333. 27318871 10.1016/j.tice.2016.05.008

[32] Wang J, Zou W, Ma J, Liu J (2019) Biomaterials and Gene Manipulation in Stem Cell-Based Therapies for Spinal Cord Injury. Stem Cells Dev 28: 239–257. 30489226 10.1089/scd.2018.0169

[33] McDonald JW, Liu XZ, Qu Y, Liu S, Mickey SK, (1999) Transplanted embryonic stem cells survive, differentiate, and promote recovery in injured rat spinal cord. Nat Med 5: 1410–1412. 10581084 10.1038/70986

[34] Mendonça Marcus Vinícius Pinheiro, Larocca Ticiana Ferreira, de Freitas Souza Bruno Solano, Villarreal Cristiane Flora, Maia Silva Luiz Flávio, (2014) Safety and neurological assessments after autologous transplantation of bone marrow mesenchymal stem cells in subjects with chronic spinal cord injury. Stem Cell Res Ther 5: 126. 25406723 10.1186/scrt516 PMC4445989

[35] Drukker M (2006) Immunogenicity of embryonic stem cells and their progeny. Methods Enzymol 420: 391–409. 17161708 10.1016/S0076-6879(06)20019-3

[36] Drukker M (2004) Immunogenicity of human embryonic stem cells: can we achieve tolerance?. Springer Semin Immunopathol 26: 201–213. 15549307 10.1007/s00281-004-0163-5

[37] Bonnamain V, Neveu I, Naveilhan P (2012) Neural stem/progenitor cells as a promising candidate for regenerative therapy of the central nervous system. Front Cell Neurosci 6: 17. 22514520 10.3389/fncel.2012.00017 PMC3323829

[38] Weiss ARR, Dahlke MH (2019) Immunomodulation by Mesenchymal Stem Cells (MSCs): Mechanisms of Action of Living, Apoptotic, and Dead MSCs. Front Immunol 10: 1191. 31214172 10.3389/fimmu.2019.01191 PMC6557979

[39] Watanabe Shuji, Uchida Kenzo, Nakajima Hideaki, Matsuo Hideaki, Sugita Daisuke, (2015) Early transplantation of mesenchymal stem cells after spinal cord injury relieves pain hypersensitivity through suppression of pain-related signaling cascades and reduced inflammatory cell recruitment. Stem Cells 33: 1902–1914. 25809552 10.1002/stem.2006

